# Estimating the monetary value of health and capability well-being applying the well-being valuation approach

**DOI:** 10.1007/s10198-020-01231-7

**Published:** 2020-09-16

**Authors:** Sebastian Himmler, Job van Exel, Werner Brouwer

**Affiliations:** 1 Erasmus School of Health Policy & Management, Erasmus University Rotterdam, Rotterdam, The Netherlands; 2 Erasmus School of Economics, Erasmus University Rotterdam, Rotterdam, The Netherlands

**Keywords:** Capability approach, Economic evaluation, Value of health, Subjective well-being, Well-being valuation, I10, I30

## Abstract

**Background:**

Quality of life measures going beyond health, like the ICECAP-A, are gaining importance in health technology assessment. The assessment of the monetary value of gains in this broader quality of life is needed to use these measurements in a cost-effectiveness framework.

**Methods:**

We applied the well-being valuation approach to calculate a first monetary value for capability well-being in comparison to health, derived by ICECAP-A and EQ-5D-5L, respectively. Data from an online survey administered in February 2018 to a representative sample of UK citizens aged 18–65 was used (*N* = 1512). To overcome the endogeneity of income, we applied an instrumental variable regression. Several alternative model specifications were calculated to test the robustness of the results.

**Results:**

The base case empirical estimate for the implied monetary value of a year in full capability well-being was £66,597. The estimate of the monetary value of a QALY, obtained from the same sample and using the same methodology amounted to £30,786, which compares well to previous estimates from the willingness to pay literature. Throughout the conducted robustness checks, the value of capability well-being was found to be between 1.7 and 2.6 times larger than the value of health.

**Conclusion:**

While the applied approach is not without limitations, the generated insights, especially concerning the relative magnitude of valuations, may be useful for decision-makers having to decide based on economic evaluations using the ICECAP-A measure or, to a lesser extent, other (capability) well-being outcome measures.

**Electronic supplementary material:**

The online version of this article (10.1007/s10198-020-01231-7) contains supplementary material, which is available to authorized users.

## Introduction

Health economic evaluations are increasingly used in health care decision making. In countries like the UK and the Netherlands, specifically cost-utility analysis is a frequently applied tool to inform the allocation of scarce (health care) resources, with the aim of optimising population health [1]. In recent years it has been questioned whether health, measured for example with instruments such as EQ-5D, is the appropriate maximand in all contexts of health care delivery. Sometimes, the benefits of care interventions may not be limited to health alone, and the aim of interventions may not be to restore or improve health, but rather to maintain or increase the well-being of patients [2, 3]. The question of what we want to maximise appears especially relevant in the palliative and elderly care sectors, and in mental health and integrated social care [4, 5]. The interventions in those areas may range from pharmaceutical interventions to home care and, in the context of multi-morbidity, combinations of treatments.

As a consequence, several instruments have been put forward, aiming to measure quality of life in a broader sense, which could be applied to broaden the evaluative space of health economic evaluations [6]. In this context, some researchers focused on an operationalisation of Amartya Sen’s capability approach [7], which emphasises the importance of individuals’ ability to reach certain well-being states (capability) instead of being in these states (functioning). A prominent example is the ICEpop CAPability measure for adults (ICECAP-A), an instrument developed for assessing the capability well-being of the general adult population. The ICECAP-A measures capabilities in five dimensions with four levels each: (i) stability (ii) attachment (iii) autonomy (iv) achievement, and (v) enjoyment [8]. The measure was validated and tested in different contexts with promising results and continues to be validated further [9–14]. Moreover, it was shown that the ICECAP-A measures a broader construct and also comprises complementary information compared to common generic health utility measures like EQ-5D-3L and EQ-5D-5L [15, 16].

In the new Dutch pharmaco-economic guidelines specific attention is paid to broader outcome measures, in particular the ICECAP instruments [17]. This may not only increase their use in the context of economic evaluations of pharmaceutical and other interventions, but also brings up the issue as to how the results of such broader economic evaluations should be used in decision making. Indeed, the current (applications of) capability measures still raise important questions [18], including how results from economic evaluations using capabilities, likely in the form of incremental cost-effectiveness ratios (ICERs), should be interpreted. Valuable in this context would be information on an appropriate threshold value for capabilities, analogous to the quality-adjusted life-year (QALY) threshold for health gains. While the monetary value of a QALY has been extensively studied, primarily using willingness to pay [19, 20], research on the monetary value of capability well-being is still lacking.

This study aims to fill this gap, by estimating a first monetary value of a year in full capability well-being, using the well-being valuation method to ICECAP-A index values in a representative sample of UK citizens aged 18–65. Using the same approach and sample, we furthermore provide estimates of the same kind for the monetary value of a QALY based on EQ-5D-5L data, facilitating a first comparison of the societal valuations of these constructs.

## Methods

### Conceptual model

The well-being valuation approach uses observational data to assess the experienced average impact of a change in a good on individuals’ overall utility *u*, proxied by subjective well-being (SWB) or life satisfaction, and calculating the change in income necessary to maintain the same level of utility [21]*.* This obtained monetary valuation is also known as compensating surplus (CS). This regression-based approach circumvents the inherent drawbacks of willingness to pay experiments by not directly asking individuals for a monetary value of a certain good [22, 23]. Applying the well-being valuation approach for estimating monetary values of capability well-being and health requires the following assumption about the relationship between health, capability and SWB: Individual's overall utility *u*, as proxied by SWB, is a function of health or capability well-being *Q*. Imposing this type of relationship on capabilities is in conflict with the normative position that capabilities go not only beyond health but also beyond utility and SWB [24]. While we do acknowledge that there is some evidence based on individual-level data in favour of this competing interpretation [25], this is a necessary assumption due to the mechanics of the well-being valuation approach.

1 $$u\left( {Q,Y,X} \right) = SWB\left( {Q,Y,X} \right).$$

Utility *u* is furthermore determined by income *Y,* and certain individual and socioeconomic characteristics summarised in vector *X.* We followed a three-stage well-being valuation procedure, as previously formulated [21, 26]. The three steps include separately estimating the impact of income and the good to be valued on SWB (steps 1 and 2) and then calculating the compensating surplus (CS) according to Eq. (2) (step 3):

2 $${\text{CS}} = Y^{0} - e^{{\left[ {\ln \left( {Y^{0} } \right) - \frac{{Q^{\prime}}}{{Y^{\prime}}}} \right]}} ,$$

*Yʹ* and *Qʹ* are the marginal effects of changes in income and health or capability on SWB, and ${Y}^{0}$ represents a representative level of population income.

### Data and model specification

The data for the analysis originated from a cross-sectional survey of UK citizens, which was not specifically designed for this analysis and is, therefore, limited to individuals aged 18–65. Random sampling and survey administration were conducted by Survey Sampling International in February 2018 using an online survey format. The sample was aimed to be representative regarding age, gender and level of education and consisted of 1512 individuals. The survey included inter alia questions about health, well-being, income, employment and marital status, religiosity and information about the health risk attitude of respondents (in the listed order) [27].

The impact of health *H* and capability well-being CW on SWB were estimated separately, due to their substantial overlap and likely collinearity. While it has been discussed before that estimating the effect of health on SWB is prone to issues of endogeneity [28, 29], it was not possible to address this issue adequately due to the limitations of the used data. Applying a previously used instrument for health—average health per socioeconomic cell—was not feasible, possibly a result of the small sample size [30], ${\mathrm{SWB}}_{i}$ was assessed using Cantril’s ladder, a one-dimensional life satisfaction instrument asking respondents to rate their life from worst possible to best possible life on a 0–10 scale [31]. The impact of health and capability well-being were estimated using ordinary least squares, assuming cardinality in the responses [32]:

3 $${\text{SWB}}_{i} = \beta_{0} + \beta_{1} H_{i} + \beta_{2} \ln \left( {Y_{i} } \right) + \beta_{3} X_{i} + \varepsilon_{i} ,$$

4 $${\text{SWB}}_{i} = \alpha_{0} + \alpha_{1} CW_{i} + \alpha_{2} \ln \left( {Y_{i} } \right) + \alpha_{3} X_{i} + \mu_{i} .$$

Health of respondents ${H}_{i}$ was measured via EQ-5D-5L utilities, applying the English EQ-5D-5L tariff estimated by Devlin et al. (2018) [33]. Capability well-being, ${\mathrm{CW}}_{i},$ was assessed via ICECAP-A index values [8, 34]. Estimates for income ${Y}_{i}$ were obtained by asking respondents to place their combined monthly household income before taxes into 12 prespecified intervals. In a follow-up question, respondents were asked to indicate exact amount within these intervals. Missing exact income amounts were imputed based on the sample means of the income interval selected in the first step, if applicable. ${X}_{i}$ contains age, gender, education, marital status, and employment status, which have been shown to influence SWB [35]. Following further guidance from the literature, we also controlled for religiosity, measured by asking for the importance of religion on a 7-point Likert-scale, and religious affiliation [36]. Information on the health risk attitude of individuals [27] was included to partly account for personality [37].

Income coefficient estimates in SWB regressions are likely endogenous due to reverse causality [38, 39], measurement error or omitted variables like working hours, or time spent away from family [40]. Instrumental variable (IV) approaches have been used to overcome this problem [41, 42]. We, therefore, applied a two-stage least squares (2SLS) approach [43], testing different available candidate instruments. In the final analysis, we used whether a household currently holds home contents insurance (CI) as an instrument for income *Y*. The logarithmic transformation of income was used to account for its diminishing marginal return on SWB [44]. The 2SLS approach took the following form:

5 $${\text{SWB}}_{i} = \gamma_{0} + \gamma_{1} H_{i} + \gamma_{2} \ln \left( {Y_{i} } \right) + \gamma_{3} X_{i} + \omega_{i} ,$$

6 $${\ln}(Y_{i} ) = \delta_{0} + \delta_{1} {\text{CI}}_{i} + \delta_{2} X_{i} + v_{i} .$$

To be a suitable instrument, CI must be sufficiently correlated with income. Possible channels could be that purchasing the insurance is more affordable if income is higher, or that higher income could lead to the household containing more valuable objects, which increases the likelihood of obtaining CI.

The instrument should furthermore only be correlated with SWB through income. However, this is generally not testable [43]. It is unlikely that the presence of contents insurance (directly) influences individuals’ SWB. The insurance effect of increased (financial) stability could be a possible channel. However, we found only a small and negative correlation between CI and the stability dimension of the ICECAP-A (*r* = − 0.15). Maintaining CI could relate to personality traits like risk aversion, which might influence SWB. Nevertheless, we were directly controlling for risk attitude, which is furthermore merely weakly correlated with CI (*r* = 0.14). Additionally, the obtained SWB values might not originate from the same individual, who decided about purchasing CI. Unfortunately, we had no information available to investigate this. Finally, CI could be indicative of possessing more valuable items or living in a nicer home, which does impact SWB [35]. However, we argue that these aspects are also, at least partly, mediated through income.

Coefficient estimates from Eqs. (3) to (6) were used to calculate the compensating surplus (CS) for one QALY and one year in full capability well-being (YFC) according to the following equations:

7 $${\text{CS}}\left( {{\text{QALY}}} \right) = \frac{1}{{{\Delta }H}}*\left[ {Y^{0} - e^{{\left[ {\ln \left( {Y^{0} } \right) - \frac{{\beta_{1} }}{{\gamma_{2} }}*{\Delta }H} \right]}} } \right],$$

8 $${\text{CS}}\left( {{\text{YFC}}} \right) = \frac{1}{{\Delta {\text{CW}}}}*\left[ { Y^{0} - e^{{\left[ {\ln \left( {Y^{0} } \right) - \frac{{\alpha_{1} }}{{\gamma_{2} }}*\Delta {\text{CW}}} \right]}} } \right].$$

where ${Y}^{0}$ was set to the sample’s median yearly household income of £27,000, while ${\Delta \mathrm{H}}$ and $\Delta \mathrm{CW}$ represented incremental changes in health and capability well-being. It was necessary to impose incremental changes of *H* and CW since under the framework laid out in Eq. (2) the CS would be constrained at the pre-specified level of income [26]. The incremental approach mirrors contingent valuation studies, where willingness to pay for small health changes are aggregated to a full QALY [19]. The size of the incremental change $\Delta$ was set to 0.1, corresponding to half a standard deviation, which was found to be a reasonable approximation of the minimally important clinical difference for health-related quality of life measurements [45].

Descriptive and regression analyses were performed using STATA 15.0 (Stata Corp. 2018. Stata Statistical Software: Release 15. College Station, TX: Stata Corp LP). 2SLS estimates were obtained using the ivreg2 package [46]. All monetary amounts presented in the following correspond to 2018 prices.

### Robustness checks

The robustness of the estimates was examined testing the following specifications: First, to gain insights into the relevance of accounting for the endogeneity of income, the non-instrumented, standard OLS income estimate was used instead of the IV income estimate. Second, an income coefficient estimate from a study based on much richer data was used. We linearly rescaled the dependent variable from 0–10 to 1–7 interval to match the SWB measure used in the analysis by Fujiwara [26], and applied his log-income coefficient estimate, as it was based on (random) lottery wins. Third, SWB was assessed via the multidimensional Satisfaction with Life Scale (SWLS) instead of Cantril’s ladder [47], with SWLS scores rescaled from 0 to 10 to facilitate comparison of coefficients. Fourth, the unweighted average of Cantril’s ladder and SWLS on a 0–10 scale were used as a compound SWB measure, as it was previously suggested that such a compound measure could be more robust than either of the measures on its own [48]. Fifth, instead of using the weighted population tariffs for scoring EQ-5D-5L and ICECAP-A values, we used the unweighted and rescaled (0–1) sum scores of these measures to test the sensitivity of the estimates to applying population tariffs, as both tariffs were based on different valuation methods. In the sixth robustness check, the mapped EQ-5D-3L value set was used instead of the EQ-5D-5L value set, since the methodology applied for the latter has come under scrutiny [49]. In the seventh specification, ${Y}^{0}$ was set to the mean yearly income of £37,843, instead of the median income of £27,000. In the last two robustness checks, $\Delta H$ and $\Delta \mathrm{CW}$ were set to 0.05 and 0.20, as the size of the increment may still be considered somewhat arbitrary.

## Results

### Estimates for income, health and capability well-being

After excluding 139 observations with no income information, and imputing income interval sample means for 358 respondents who only reported their income interval, the analysis sample included 1373 individuals. There were no missing values in the remaining variables. This sample was comparable to the UK population aged 18–65 concerning most characteristics (Table 1). The reported average yearly gross income of £37,843 in the sample is lower than the UK average of £45,773 in 2018. The average ICECAP-A index is slightly lower than previously observed in a general population sample, which included individuals above 65 with generally lower capabilities [50].

**Table 1:** Characteristics of analysis sample and IV-sample

	Total sample		IV-sample	
	Mean	SD	Mean	SD
Cantril’s ladder	6.4	2.0	6.9	1.8
ICECAP-A	0.75	0.20	0.79	0.176
EQ-5D-5L	0.84	0.21	0.85	0.205
HH income in £	37,843	56,729	45,200	78,838
Age	42.6	13.9	47.2	12.4
Female	51.8%		48.8%	
Tertiary education	45.4%		50.0%	
Marital status				
Married	59.5%		66.8%	
Divorced/widowed	9.2%		10.9%	
Never married	31.3%		22.3%	
Employment status				
Employed	54.8%		61.9%	
Self-employed	9.5%		9.2%	
Unemployed	5.5%		2.2%	
Homemaker	9.7%		6.1%	
Student	5.2%		1.0%	
Retired	9.5%		14.8%	
Unable to work	5.8%		4.9%	
Religious affiliation				
Christian	42.1%		49.8%	
Atheist	32.8%		29.5%	
Agnostic	13.0%		11.9%	
Muslim	3.8%		1.8%	
Other religion	8.4%		7.0%	
Importance of religion	2.8	2.0	2.8	2.1
HRAS	29.0	5.8	30.1	5.4
*N*	1373		1373	

*IV* instrumental variable, *HH* household; Importance of religion measured on a 1 (low) to 7 (high) scale; *HRAS* health risk attitude scale ranging from 6 (risk loving) to 42 (risk averse)

Coefficients from the separate health and capability regressions as described in Eqs. (3) and (4) are shown in columns (I) and (II) of Table 2. Parameters estimates for EQ-5D-5L and ICECAP-A were positive and significant, (2.665 and 6.234), meaning that health and capability have the expected positive impact on SWB. The signs of the coefficients of most control variables corresponded to findings from the literature [35, 41]. Coefficient estimates from the 2SLS IV regression are shown in column (III). Around a third of respondents (*N* = 516) reported that their household holds contents insurance. The log-income coefficient was 2.201. Control variables deviated slightly between the models, namely in a higher positive impact of being retired, a negative impact of education and no effect of marital status and unemployment.

**Table 2:** Results of OLS and IV regressions

	(I)		(II)		(III)	
	Health		Capability		Income-IV	
Log yearly income	0.495***	(0.065)	0.308***	(0.054)	2.201***	(0.638)
EQ-5D-5L	− 2.665***	(0.305)			2.310***	(0.378)
ICECAP-A			6.234***	(0.243)		
Age	**− **0.026	(0.029)	**− **0.006	(0.024)	**− **0.004	(0.037)
Age-squared	0.0003	(0.000)	0.0001	(0.000)	0.0001	(0.000)
Male	**− **0.011	(0.093)	**− **0.012	(0.075)	**− **0.068	(0.119)
Tertiary education	0.038	(0.094)	**− **0.085	(0.076)	**− **0.395*	(0.199)
Divorced or widowed	**− **0.358*	(0.168)	0.078	(0.132)	0.256	(0.304)
Never married	**− **0.536***	(0.121)	**− **0.033	(0.096)	0.202	(0.306)
Self-employed	0.100	(0.180)	0.117	(0.139)	0.451	(0.249)
Unemployed	**− **0.579*	(0.231)	**− **0.275	(0.190)	0.661	(0.546)
Homemaker	**− **0.257	(0.169)	**− **0.028	(0.133)	0.387	(0.308)
Student	**− **0.357	(0.247)	**− **0.589**	(0.226)	**− **0.084	(0.365)
Retired	0.537**	(0.188)	0.115	(0.148)	0.864***	(0.253)
Unable to work	**− **0.514	(0.277)	**− **0.541**	(0.197)	0.672	(0.534)
Atheist	0.245	(0.138)	0.182	(0.111)	0.268	(0.168)
Agnostic	0.097	(0.161)	0.095	(0.139)	0.038	(0.202)
Muslim	**− **0.462	(0.303)	0.013	(0.241)	**− **0.330	(0.307)
Other religion	**− **0.013	(0.172)	0.101	(0.145)	**− **0.095	(0.235)
Importance of religion	0.147***	(0.031)	0.097***	(0.025)	0.148***	(0.038)
HRAS	0.077***	(0.009)	0.033***	(0.007)	0.0687***	(0.011)
Constant	**− **2.858**	(0.954)	**− **2.657***	(0.767)	**− **20.60**	(6.687)
*N*	1373		1373		1373	
Root MSE	1.662		1.345		2.021	
*R*-squared	0.334		0.564		–	
Kleibergen-Paap rk LM					21.55***	
Kleibergen-Paap rk Wald F					21.63***	
Test for endogeneity					10.65***	

Input parameters for Eqs. (7) and (8) are in bold

*HRAS* health risk attitude scale; Standard errors in parentheses; *p < 0.05, **p < 0.01, ***p < 0.001

Kleibergen-Paap rk Wald F statistic (21.832 with Stock-Yogo critical 10% value 16.38) and Kleibergen-Paap rk LM statistic of (21.746, *p* < 0.001), indicated that the used instrument was not weak or underidentified. This was further substantiated by a significant coefficient (*p* < 0.001) of CI in the first stage regression (Appendix A). The characteristics of the IV sample were reasonably similar to the full sample (Table 1), with slightly higher levels of life satisfaction, capability well-being and income. Testing for the endogeneity of log income revealed that the variable should not have been treated as exogenous (*p* < 0.001).

### Implied monetary values and results from robustness checks

The resulting monetary valuations of one QALY and one YFC were £30,786 and £66,597, respectively. The relative size of the monetary value of capability well-being compared to health was thereby estimated to be 2.2. Coefficients estimates and the corresponding monetary valuations for the conducted robustness checks are shown in Table 3. First, not instrumenting for income led to considerably larger monetary estimates of one QALY (£112,336) and one YFC (£193,305). Second, applying the income coefficient from Fujiwara (2013), who used lottery wins, led to slightly higher monetary estimates compared to the base case. Third, using SWLS instead of Cantril’s ladder provided an almost identical monetary value for one YFC, while the value of one QALY was reduced to £20,988. Fourth, the use of the compound SWB score averaged out differences in coefficients and monetary valuations between the use of Cantril’s ladder and SWLS as SWB proxies. Fifth, employing sum scores of EQ-5D-5L and ICECAP-A resulted in slightly higher estimates of the value of one QALY and conversely, slightly lower estimates for one YFC. Applying the mapped EQ-5D-3L tariff reduced the monetary valuation of one QALY to £25,487. In the last three robustness tests, the income model had to be recalculated. As in the base case, the instrument passed under- and weak identification tests. Seventh, replacing median income by mean income increased the valuations to £43,149 and £93,343, respectively. Altering the imposed incremental change of 0.1 index points to 0.05 reduced the monetary estimates slightly while imposing a 0.2 incremental change led to higher estimates compared to the base case. Throughout model alterations, the monetary equivalent value of one YFC exceeded that of one QALY by a factor of around two, with the robustness check utilising SWLS as SWB proxy as an outlier.

**Table 3:** Base case monetary estimates and robustness to alternative specifications

	Base case	No IV	IV Fujiwara^a^	SWLS^b^	Compound SWB^c^	Sum scores^d^	EQ5D-3L tariff	Mean income	Increment 0.05	Increment 0.20
Coefficients										
Log income	2.201	0.495	1.103	2.633	2.417	2.255	2.197	Base case coefficients		
EQ-5D-5L	2.665	2.665	1.599	2.131	2.398	2.858	2.179			
ICECAP-A	6.234	6.234	3.740	7.488	6.861	5.881	6.234			
Value in £										
1 QALY	30,786	112,336	36,431	20,988	25,498	32,141	25,487	43,149	31,717	29,031
1 YFC	66,597	193,305	77,651	66,828	66,723	61,979	66,597	93,343	71,305	58,384
Rel. size	2.2	1.7	2.1	3.2	2.6	1.9	2.6	2.2	2.2	2.0

All reported coefficients significant on the 1% level

^a^Income coefficient from Fujiwara et al. (2013) [26], other coefficients from rerunning regressions with rescaled SWB

^b^Rescaled from 0 to 10, instrument passes under- and weak identification test

^c^Unweighted average of Cantril’s ladder and SWLS as SWB proxy, instrument passes under- and weak identification test

^d^EQ-5D-5L and ICECAP-A sum scores scaled from 0 to 1

## Discussion

### Findings and related literature

Applying the well-being valuation method, we obtained a first estimate of the monetary value of ICECAP-A-derived capability well-being for the UK. We furthermore calculated the monetary value of health and were able to compare the valuations of one QALY and one YFC directly. The empirical challenge inherent to the chosen approach is the endogeneity of income, which we tried to overcome using whether a household holds contents insurance as an instrument for income. In the base case model specification, this yielded monetary valuations of £30,786 for one QALY and £66,597 for one YFC, corresponding to a ratio of 2.2. The conducted robustness checks produced relative magnitudes of these monetary valuations ranging from 1.7 to 2.6.

The calculated monetary value of a QALY lies within the range of estimates from the international willingness to pay literature, which on aggregate produced a trimmed mean and median estimate of £63,777 and £20,834 (in 2010 lb) [19]. UK specific estimates from Mason et al. (2009) and Baker et al. (2010) ranged from £24,219 to £70,896 and £16,000 and £24,805 (in 2010 lb), respectively [51, 52]. In the only other application of the well-being valuation method for this purpose to date, the monetary value of one QALY in Australia was estimated to be A$42,250 (£20,797) and A$67,022 (£32,990) for short and long-term health gains using 2015 prices [41]. The relative size of the reported monetary value of well-being (A$112,000 or £55,130) compared to one QALY was 1.7, not dissimilar to what we observed in our analysis.

### Limitations

Although our results appear to have some face validity and are reasonably robust to model specifications, we need to acknowledge several limitations. On a more conceptual level, the chosen approach relies on the assumption that SWB is an appropriate proxy for individuals’ utility. This may be a strong assumption, as SWB (or happiness) is not the only thing that people care about and preferences outside of SWB maximisation exist [21]. Nevertheless, based on the findings from subjective well-being research, as for example summarised by Diener et al. (2018) [53], we argue that SWB matters enough to be able to use it as a proxy for welfare. At the same time, we must acknowledge that the validity and reliability of SWB measures have been questioned before. These concerns were addressed in detail for example by Veenhoven (2012) [54]. What we can infer from our own analysis, is that the choice of SWB instrument does have an impact on the monetary estimates (Table 3), although observed differences were not substantial. The SWLS appears to capture a different part of SWB than Cantril’s ladder does. Differing results are likely a consequence of the SWLS containing two questions, which are more related to the past (“So far I have gotten the important things I want in life” and “If I could live my life over, I would change almost nothing”), while Cantril’s ladder only asks about SWB at present, which is more consistent with the present based well-being valuation approach [47]. The well-being valuation literature so far does not provide guidance on the appropriateness of one- or multi-dimensional SWB measures, or the use of a composite of both. This should be examined in future research.

A further limitation is that we had to deviate from the intended three-stage well-being valuation approach in two ways [21, 26]: First, including control variables in order to prevent omitted variable bias conflicts with the idea of using total causal effects in calculating the monetary valuations as outlined before [21]. In the analysis by Fujiwara (2013), the difference in unemployment coefficients between a model without any covariates and a model controlling for several variables was minimal (− 0.441 and − 0.436). Removing all control variables from models (I) to (III) generated monetary estimates for one QALY and one YFC of £33,914 and £63,156, respectively, close to the base case estimates. Second, and potentially more problematic, we assumed exogeneity of both health and capability well-being due to the lack of suitable instruments. When health was instrumented in a previous analysis, the estimated impact of a change in health decreased slightly [30]. Assuming this would also hold in our context, our monetary valuations represent overestimations.

It is furthermore inherently difficult to demonstrate that the used income instrument (contents insurance) satisfies the exclusion restriction assumption. In the second robustness check, we employed the log-income coefficient of Fujiwara (2013) for the UK, as an external reference point, after basing the analysis on the same SWB scale [26]. While not without limitations, his estimate, based on large scale panel data and exploiting random income shocks like lottery wins, can be considered as close to causal estimates as it gets when using non-experimental data. The reported log-income coefficient of 1.103 is comparable to the estimate we obtained when repeating the analysis on the same SWB scale of 1.321. Monetary estimates increased by around 20% (Table 3). Judging from this comparison, it appears that our instrument performs reasonably well.

The extent to which our results are generalisable to the general UK population is unclear, as our sample did not include individuals aged 65 and above. Previous research suggests that functional limitations and social functioning, which are more related to the ICECAP-A, could be more relevant to the elderly than typical health dimensions, like morbidities or pain [55]. To test this, we included an interaction term for the respective quality of life index and age to the base case models. We observed a positive and significant coefficient of 0.031 (*p* = 0.042) for an interaction term between ICECAP-A and age, while the interaction coefficient of EQ-5D-5L and age of 0.021 was not significant (*p* = 0.355). This indicates that omitting the elderly may have introduced a downward bias for the value of one YFC in comparison to the value of one QALY. Furthermore, due to relying on data from online survey panels, the individuals in the sample, in general, were quite healthy, with an average EQ-5D-5L index of 0.837 (SD 0.21). We do not know how the lack of sufficient observations at the lower end of the scale influenced our overall results. Lastly, we lacked information on the household size of respondents, which precluded the use of equivalised household income, to facilitate the comparability across household compositions [40, 41].

### Interpretation and implications of the results

While the calculated values for one QALY and one YFC varied across the conducted robustness checks, their ratio fluctuated at around two. As well-being measures were designed to capture quality of life beyond health, it is explicable that the monetary value of well-being in general lies above the value of health alone. That this also holds for capability well-being could have been expected but had not yet been confirmed before.^1^ This information is relevant in the context of interpreting results of economic evaluations using broader outcome measures, which may be relevant in a range of interventions (from pharmaceuticals to palliative care) that have benefits not fully captured in conventional QALY measures.

The interpretation of the relative magnitude of the monetary estimates of one QALY and one YFC deserve further attention, considering that the EQ-5D-5L and the ICECAP-A are anchored on two different scales. The former is anchored on a 0 to 1, dead to full health scale, with the possibility of health states below zero [33]. The latter ranges from 0 to 1 for no capability to full capability, where death implies no capabilities, but no capabilities, in turn, does not necessarily imply death [34, 56]. While it is plausible that on the higher end of the scale, capabilities go beyond health on an underlying overall quality of life continuum, it is less clear on the lower end of the scale, as having no capabilities could be equivalent to death, but also lower or higher in terms of overall quality of life. This may have implications for the comparability of the monetary valuations, as the imposed incremental change in health and capability of 0.1 may represent either a larger or smaller difference in the underlying utility. Future research could investigate these issues further, for instance, by focusing on the behaviour of SWB scores at very low levels of capabilities and health.

If capability well-being, as measured by the ICECAP-A, is included in future economic evaluations in areas where a focus on health is potentially too restrictive to capture all relevant benefits of an intervention, the here presented results could give a first indication about a cost-effectiveness threshold. In practice, ICERs calculated using ICECAP-A index values could be compared to the here estimated monetary value of a YFC. Our estimates are especially relevant for countries that relate their threshold to the societal monetary value of health or wellbeing gains, like the Netherlands [20]. In other countries, like the UK, thresholds are conceptually more related to the marginal cost-effectiveness of current spending [57]. Conceptually, this limits the direct applicability of our results in the UK, while it is noteworthy that obtaining opportunity cost based monetary estimates for capability well-being seems to be a challenging task.

Future research should aim for confirming our findings for the absolute and relative monetary valuation of capability well-being in general, either by employing alternative approaches, like willingness to pay or discrete choice experiments or by applying the well-being valuation method to other, preferably richer data sets. Prerequisite for the latter should be the availability of potential instruments for income. On a different note, while there are first applications, more conceptual and theoretical work is needed about whether, when and how capability well-being should be included in health economic evaluations [58]. One open question for example is, whether full capability or a sufficient level of capability, which was established recently, should be considered as the objective of interventions [59]. Nevertheless, and to conclude, the results of our analysis may be useful as a first estimate of a threshold value for a YFC that can be used when making decisions based on economic evaluations using the ICECAP-A, or to a lesser extent, other (capability) well-being outcome measures.

## Electronic supplementary material

Below is the link to the electronic supplementary material.

Supplementary file 1 (DOCX 31 kb)

## Data Availability

The data used in this study is not proprietary and can be made available upon request.

## Footnotes

1 At the same time, it may be more difficult to achieve similarly sized increases on these broader measures.
