# Supplementary material for: Estimating the monetary value of health and capability well-being applying the well-being valuation approach
Source: Eur J Health Econ. 2020 Sep 16;21(8):1235–44. doi: 10.1007/s10198-020-01231-7 (PMC7561589; doi:10.1007/s10198-020-01231-7)
Supplement: Supplementary file 1 — (DOCX 31 kb) [file 10198_2020_1231_MOESM1_ESM.docx]

**Appendix**

**Appendix A** First Stage regression results

|  |  |  |
| --- | --- | --- |
|  | First stage |  |
| Contents insurance | -0.181^***^ | (0.039) |
| EQ-5D-5L | -0.202 | (0.105) |
| Age | -0.016 | (0.013) |
| Age-squared | -0.0001 | (0.0001) |
| Male | -0.028 | (0.040) |
| Tertiary education | -0.246^***^ | (0.039) |
| Divorced or widowed | -0.353^***^ | (0.068) |
| Never married | -0.424^***^ | (0.046) |
| Self-employed | -0.188^**^ | (0.071) |
| Unemployed | -0.693^***^ | (0.108) |
| Homemaker | -0.340^***^ | (0.060) |
| Student | -0.132 | (0.136) |
| Retired | -0.201^**^ | (0.072) |
| Unable to work | -0.677^***^ | (0.101) |
| Atheist | -0.005 | (0.055) |
| Agnostic | -0.046 | (0.069) |
| Muslim | -0.052 | (0.098) |
| Other religion | -0.058 | (0.081) |
| Importance of religion | -0.0002 | (0.013) |
| HRAS | -0.004 | (0.003) |
| Constant | -10.444^***^ | (0.311) |
| N | 1,373 |  |

*Note.* HRAS, Health Risk Attitude Scale;

Standard errors in parentheses; ^*^ *p* < 0.05, ^**^ *p* < 0.01, ^***^ *p* < 0.001.
